# Supplementary material for: A co-transcriptional ribosome assembly checkpoint controls nascent large ribosomal subunit maturation
Source: Nat Struct Mol Biol. 2023 Apr 10;30(5):594–9. doi: 10.1038/s41594-023-00947-3 (PMC10191850; doi:10.1038/s41594-023-00947-3)
Supplement: Supplementary file 1 — Supplementary Table 1 and Supplementary Figure 1 [file 41594_2023_947_MOESM1_ESM.pdf]

nature structural & molecular biology

Article

https://doi.org/10.1038/s41594-023-00947-3

A co-transcriptional ribosome assembly checkpoint controls nascent large ribosomal subunit maturation

In the format provided by the authors and unedited

Supplementary Information

Supplementary Table 1. Molecular models of the Noc1-Noc2 RNP.

Supplementary Figure 1. Uncropped Northern blot images with densitometry analysis

Supplementary Table 1.

Subgroup	Chain ID	SegID	Molecule name	Total residues or bases	Modelled (residue range)	Initial PDB template
RNA	1	L1	25S	3,396	atomic (1-35, 51-131, 136-168, 251-280, 286-337, 378-393, 407-446, 489-705, 721-751, 778-801,941-952, 1161-1195, 1310-1443)	6C0F
	2	L2	5.8S	158	atomic (1-16, 24-96, 111-158)	6C0F
	3	L3	ITS2	232	atomic (1-67, 213-232)	6C0F
Ribosomal proteins	C	LC	Rpl4A_uL4	362	atomic (2-46, 110-347)	6C0F
	E	LE	Rpl6A_eL6	176	atomic (7-107, 135-176)	6C0F
	e	SE	Rpl32_eL32	130	atomic (52-129)	6C0F
	F	LF	Rpl7A_uL30	244	atomic (3-244)	6C0F
	f	SF	Rpl33A_eL33	107	atomic (4-18, 24-52, 64-107)	6C0F
	G	LG	Rpl8A_eL8	256	side-chain trimmed (53-239)	6C0F
	i	SI	Rpl36A_eL36	100	side-chain trimmed (25-100)	6C0F
	L	LL	Rpl13A_eL13	199	side-chain trimmed (52-127)	6C0F
	M	LM	Rpl14A_eL14	138	atomic (11-125)	6C0F
	N	LN	Rpl15A_eL15	204	side-chain trimmed (2-68, 96-176)	6C0F
	O	LO	Rpl16A_uL13	199	atomic (3-58, 73-199)	6C0F
	Q	LQ	Rpl18A_eL18	186	atomic (15-146)	6C0F
	S	LS	Rpl20A_eL20	172	atomic (2-167)	6C0F
Assembly factors	K	LK	Cic1	376	side-chain trimmed (31-51, 71-303)	6C0F
	n	SN	Nop7	605	side-chain trimmed (13-43, 61-267, 351-460)	6C0F
	o	SO	Nop15	220	side-chain trimmed (88-220)	6C0F
	t	ST	Rlp7	322	side-chain trimmed (54-105, 127-214, 227-322)	6C0F
	s	SS	Erb1	807	side-chain trimmed (239-310, 331-395)	6C0F
	D	LD	Mak16	306	atomic (2-131, Zn-308)	6C0F
	p	SP	Has1	505	side-chain trimmed (42-252, 264-489)	6C0F
	b	SB	Brx1	291	side-chain trimmed (31-194, 211-263)	6C0F
	m	SM	Ebp2	427	side-chain trimmed (196-257)	6C0F
	z	SZ	Rrp1	278	atomic (1-186, 197-253)	6C0F
	5	L5	Noc1	1025	atomic (213-503, 550-701, 744-795, 832-866)	de novo/Alphafold
	6	L6	Noc2	710	atomic (175-215, 220-692)	de novo/Alphafold
Metal ions	Y	LY	Magnesium	33		6C0F

Supplementary Table 1. Molecular models of the Noc1-Noc2 RNP.

Individual protein and RNA chains are listed with their initial PDB template (if not built *de novo*), and clustered into categories of RNA, ribosomal proteins and assembly factors.

Supplementary Figure 1.

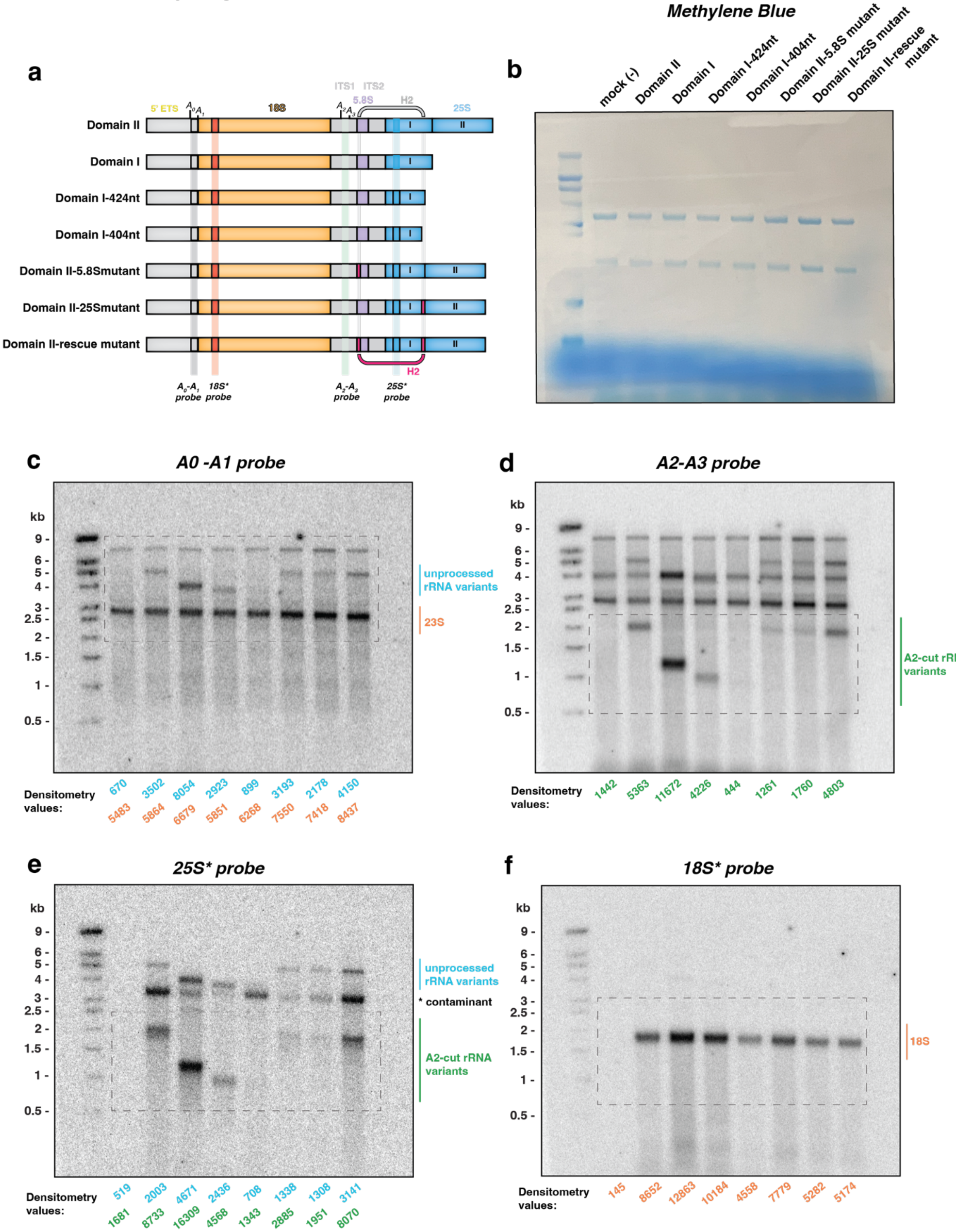

Supplementary Figure 1. Northern blot analysis of rRNA variants emphasize significance of helix 2.

(a) A schematic of plasmids used for the experiment, containing the entire SSU rRNA (5'ETS, 18S), plus various truncated forms of the LSU rRNA (Domain II, Domain I, Domain I-424nt and Domain I-404nt), including mutations to disrupt or rescue helix 2 formation (Domain II-5.8S mutant, Domain II-25S mutant and Domain II-rescue mutant). WT helix 2 is indicated in gray and the mutations to helix 2 are marked in magenta. The positions of the Northern blot probes used in (c-f) are indicated and color-coded on the plasmid schematic. **(b)** Whole cell RNA extracted from yeast cells over-expressing the above plasmids were quantified and loaded onto an RNA gel, stained with methylene blue dye (uncropped), with mock (untransformed yeast, negative control) in the first lane. **(c-f)** Uncropped northern blot analysis images of the transferred total extracted RNA probed with **(c)** A0-A1 probe, **(d)** A2-A3 probe, **(e)** 25S* specific probe and **(f)** 18S* specific probe. Northern blot experiments were repeated independently (n=2) with similar results. Dotted line boxes indicate the cropped images shown in Figure 3. Densitometry analysis of the indicated bands are color coded and numerical values are shown below each of the blots.
