# Supplementary material for: A co-transcriptional ribosome assembly checkpoint controls nascent large ribosomal subunit maturation
Source: Nat Struct Mol Biol. 2023 Apr 10;30(5):594–9. doi: 10.1038/s41594-023-00947-3 (PMC10191850; doi:10.1038/s41594-023-00947-3)
Supplement: Supplementary file 2 — Reporting Summary [file 41594_2023_947_MOESM2_ESM.pdf]

nature portfolio

Corresponding author(s): Sebastian Klinge

Last updated by author(s): 02/14/2023

Reporting Summary

Nature Portfolio wishes to improve the reproducibility of the work that we publish. This form provides structure for consistency and transparency in reporting. For further information on Nature Portfolio policies, see our Editorial Policies and the Editorial Policy Checklist.

Statistics

For all statistical analyses, confirm that the following items are present in the figure legend, table legend, main text, or Methods section.

n/a	Confirmed	
☐	☒	The exact sample size (*n*) for each experimental group/condition, given as a discrete number and unit of measurement
☐	☒	A statement on whether measurements were taken from distinct samples or whether the same sample was measured repeatedly
☐	☒	The statistical test(s) used AND whether they are one- or two-sided<br>*Only common tests should be described solely by name; describe more complex techniques in the Methods section.*
☒	☐	A description of all covariates tested
☐	☒	A description of any assumptions or corrections, such as tests of normality and adjustment for multiple comparisons
☐	☒	A full description of the statistical parameters including central tendency (e.g. means) or other basic estimates (e.g. regression coefficient) AND variation (e.g. standard deviation) or associated estimates of uncertainty (e.g. confidence intervals)
☐	☒	For null hypothesis testing, the test statistic (e.g. *F*, *t*, *r*) with confidence intervals, effect sizes, degrees of freedom and *P* value noted<br>*Give P values as exact values whenever suitable.*
☒	☐	For Bayesian analysis, information on the choice of priors and Markov chain Monte Carlo settings
☒	☐	For hierarchical and complex designs, identification of the appropriate level for tests and full reporting of outcomes
☒	☐	Estimates of effect sizes (e.g. Cohen's *d*, Pearson's *r*), indicating how they were calculated

Our web collection on statistics for biologists contains articles on many of the points above.

Software and code

Policy information about availability of computer code

Data collection	Cryo-EM datasets were collected using SerialEM (v. 3.8) on an FEI Titan Krios (FEI/Thermofisher) microscope operating at 300 kV and 22,500x magnification. 8 datasets (Noc1-Noc2 RNP) and 2 datasets (Noc2-Noc3 RNP) were collected at super-resolution pixel size of 0.65 Å/px, with defocus values ranging from -1.0 to -3.0 µm. The total electron dose was 37.9 electrons/Å2 over 32 frames. For comparative IP-MS/MS experiments, samples were analyzed by reversed phase nano-LC-MS/MS using a Fusion Lumos (Thermo Scientific).
Data analysis	Cryo-EM movies were aligned and averaged in Relion (v. 3.0) using Relion implementation of MotionCor2-like algorithm, and Contrast transfer function parameters were estimated using CTFFIND4.1. Particles were picked using crYOLO (v. 1.3.5). Relion and cryoSPARC (v. 3.3.1) was used for subsequent classification and refinement steps. Models were manually built in Coot 0.9 using starting models from PDB (code 6C0F) and from the AlphaFold database. Refinement was performed using PHENIX 1.19. Final models were validated using MolProbity, EMRinger, and phenix.rna_validate. For comparative IP-MS/MS experiments, data were quantified and searched against the S. cerevisiae Uniprot protein database (2019) concatenated with the MS2-protein sequence and common contaminations. For the search and quantification, MaxQuant (v. 2.0.3.0) was used. Oxidation of methionine and protein N-terminal acetylation were allowed as variable modifications and all cysteines were treated as being carbamidomethylated. The 'match between runs' option was enabled, and false discovery rates for proteins and peptides were set to 1% and 2% respectively. Protein abundances were expressed as LFQ (label free quantitation) values. Data were analyzed using Perseus (v.1.6.10.50). A two-tailed Student t-test was carried out to confirm statistical significance of data and Q-values resulting from FDR-correction are provided.

For manuscripts utilizing custom algorithms or software that are central to the research but not yet described in published literature, software must be made available to editors and reviewers. We strongly encourage code deposition in a community repository (e.g. GitHub). See the Nature Portfolio guidelines for submitting code & software for further information.

Data

Policy information about availability of data

All manuscripts must include a data availability statement. This statement should provide the following information, where applicable:

- Accession codes, unique identifiers, or web links for publicly available datasets
- A description of any restrictions on data availability
- For clinical datasets or third party data, please ensure that the statement adheres to our policy

Atomic coordinates and EM maps have been deposited in the Protein Data Bank and Electron Microscopy Data Bank under the following accession codes: Noc1-Noc2 RNP (EMDB-27919, PDB 8E5T) and Noc2-Noc3 RNP (EMD-27910). Raw cryo-EM data has been uploaded to the EMPIAR database (EMPIAR-11379).
Data used but not generated in the study: Starting model for model building of the Noc1-Noc2 RNP (PDB 6C0F).

Human research participants

Policy information about studies involving human research participants and Sex and Gender in Research.

Reporting on sex and gender	N/A
Population characteristics	N/A
Recruitment	N/A
Ethics oversight	N/A

Note that full information on the approval of the study protocol must also be provided in the manuscript.

Field-specific reporting

Please select the one below that is the best fit for your research. If you are not sure, read the appropriate sections before making your selection.

☒ Life sciences ☐ Behavioural & social sciences ☐ Ecological, evolutionary & environmental sciences

For a reference copy of the document with all sections, see nature.com/documents/nr-reporting-summary-flat.pdf

Life sciences study design

All studies must disclose on these points even when the disclosure is negative.

Sample size	Sample sizes for cryo-EM datasets were not predetermined. 18,046 (Noc1-Noc2 RNP) and 8,249 (Noc2-Noc3 RNP) micrographs were collected. The number of particles extracted from these micrographs were not predetermined. For comparative IP-MS/MS experiments, three yeast strains were used in technical triplicate for purification and mass spectrometry.
Data exclusions	Mis-aligned, damaged, or contaminating particles were excluded from final reconstructions during image processing. For comparative IP-MS/MS experiments, data were filtered such that a protein must be present in all 3 replicates for at least 1 condition.
Replication	Immunoprecipitation experiments were repeated independently at least 3 times with similar results. Comparative IP-MS/MS experiments were performed in technical triplicate. Data for comparative IP-MS/MS experiments were gathered from three purification experiments, and three out of three experiments showed similar results. Northern blotting experiments were performed twice independently with similar results.
Randomization	During refinement, the gold-standard approach was used to randomly assign particles to half-sets of data that are independently averaged and compared to obtain resolution estimates. Other experiments did not require randomization.
Blinding	During single particle analysis, particles are randomly assigned into half-sets, thus no blinding is applicable. No additional grouping was applied.

Reporting for specific materials, systems and methods

We require information from authors about some types of materials, experimental systems and methods used in many studies. Here, indicate whether each material, system or method listed is relevant to your study. If you are not sure if a list item applies to your research, read the appropriate section before selecting a response.

Materials & experimental systems

n/a	Involved in the study	
☒	☐	Antibodies
☒	☐	Eukaryotic cell lines
☒	☐	Palaeontology and archaeology
☒	☐	Animals and other organisms
☒	☐	Clinical data
☒	☐	Dual use research of concern

Methods

n/a	Involved in the study	
☒	☐	ChIP-seq
☒	☐	Flow cytometry
☒	☐	MRI-based neuroimaging
